# CONUT Score Predicts Early Morbidity After Liver Transplantation: A Collaborative Study

**DOI:** 10.3389/fnut.2021.793885

**Published:** 2022-01-07

**Authors:** Gabriele Spoletini, Flaminia Ferri, Alberto Mauro, Gianluca Mennini, Giuseppe Bianco, Vincenzo Cardinale, Salvatore Agnes, Massimo Rossi, Alfonso Wolfango Avolio, Quirino Lai

**Affiliations:** ^1^General Surgery and Liver Transplantation, Fondazione Policlinico Universitario Agostino Gemelli IRCCS, Rome, Italy; ^2^General Surgery and Organ Transplantation Unit, Sapienza University of Rome, Rome, Italy

**Keywords:** nutrition, immunology, post-operative morbidity, liver transplant complications, cholesterol, albumin, lymphocyte count

## Abstract

**Introduction:** Liver transplantation (LT) is burdened by the risk of post-operative morbidity. Identifying patients at higher risk of developing complications can help allocate resources in the perioperative phase. Controlling Nutritional Status (CONUT) score, based on lymphocyte count, serum albumin, and cholesterol levels, has been applied to various surgical specialties, proving reliable in predicting complications and prognosis. Our study aims to investigate the role of the CONUT score in predicting the development of early complications (within 90 days) after LT.

**Methods:** This is a retrospective analysis of 209 patients with a calculable CONUT score within 2 months before LT. The ability of the CONUT score to predict severe complications, defined as a Comprehensive Complication Index (CCI) ≥42.1, was examined. Inverse Probability Treatment Weighting was used to balance the study population against potential confounders.

**Results:** Patients with a CCI ≥42.1 had higher CONUT score values (median: 7 vs. 5, *P*-value < 0.0001). The CONUT score showed a good diagnostic ability regarding post-LT morbidity, with an AUC = 0.72 (95.0%CI = 0.64–0.79; *P*-value < 0.0001). The CONUT score was the only independent risk factor identified for a complicated post-LT course, with an odds ratio = 1.39 (*P*-value < 0.0001). The 90-day survival rate was 98.8% and 87.5% for patients with a CONUT score <8 and ≥8, respectively.

**Conclusions:** Pre-operative CONUT score is a helpful tool to identify patients at increased post-LT morbidity risk. Further refinements in the score composition, specific to the LT population, could be obtained with prospective studies.

## Introduction

Liver transplantation (LT) is the cure for a growing number of patients with end-stage liver disease. Many patients who were once deemed too frail are now considered for LT (1). However, due to the necessity to fulfill the gap between offer and demand of liver grafts, increased utilization of extended-criteria donors has led to more risky donor-to-recipient matches (2). These challenging matches contribute to post-operative morbidity and poor long-term outcomes (3).

With the intent to identify frail patients with a greater post-LT risk of complications, sophisticated scores have been introduced focusing on graft function recovery and efficacious retransplantation (4, 5). Malnutrition and immunological status can influence treatment outcomes, with various studies weighing their impact after surgery (6–8). The Controlling Nutritional Status (CONUT) score has been developed to measure both aspects and has been trialed in different settings, including cancer surgery and oncologic treatments (9–12). The CONUT score has been tested with the intent to predict overall survival and hepatocellular cancer (HCC) recurrence after LT and post-operative complications in pancreatic, esophageal, gastrointestinal, and orthopedic surgery (13–16).

However, the ability of the CONUT score to predict post-LT early morbidity and mortality has not been investigated yet. The primary aim of the study was to investigate the role of the CONUT score calculated before LT in predicting the development of severe post-transplant complications as graded by the Comprehensive Complication Index (CCI). The secondary aim was to investigate the role of the pre-LT CONUT score in predicting post-operative mortality within 90 days post-LT.

## Materials and Methods

### Study Design

This is a retrospective bicentric observational study investigating the data of patients undergoing LT.

The Strengthening the Reporting of Observational Studies in Epidemiology (STROBE) guidelines were followed to create the study.

### Setting

The participant centers were Sapienza University of Rome, Umberto I Polyclinic of Rome, and Catholic Rome University, Gemelli Hospital.

### Population

A total of 209 cases transplanted at Sapienza Rome University (period January 2013–December 2020) and Catholic Rome University (period September 2016–December 2020) were considered for the analysis. The only inclusion criterion was the availability of enough data for calculating the CONUT, and the CCI scores were enrolled for the study.

All the study subjects were adult (≥18 years) patients receiving a graft from a deceased-brain donor, including split grafts and retransplants.

### Outcomes

The primary outcome of the study was the development of a complex post-operative course defined as a CCI ≥42.1. The secondary outcome was the post-LT 90-day mortality. The last follow-up date was May 31st, 2021.

### Data Collection

Data were retrospectively obtained from the prospectively collected charts of the patients. The guarantor of the data quality was the Data Manager of the Study Group (QL). Data errors and missingness were identified across the database and solved, when possible, with specific queries.

### Definitions

The CONUT score was calculated according to the original descriptions (9–12). The CONUT score is based on serum albumin, cholesterol, and total lymphocyte count. (12) CONUT score ranges from 0 (i.e., normal nutritional status) to 12 (i.e., severe malnutrition) (Table 1). The CONUT score was calculated using the last available data from blood tests of patients on the LT waitlist. We arbitrarily decided to select an upper limit of 2 months before LT for calculating the score: all the patients with data older than 2 months before the transplant were excluded from the study.

**Table 1:** Controlling nutritional status score calculation.

**Variables**	**Undernutrition status**			
	**Normal**	**Light**	**Moderate**	**Severe**
**Albumin (g/dL)**	≥3.5	3.0–3.49	2.5–2.9	<2.5
**Points**	0	2	4	6
**Total lymphocyte count (/mm** ^**3**^ **)**	>1,600	1,200–1,599	800–1,199	<800
**Points**	0	1	2	3
**Total cholesterol (mg/dL)**	>180	140–180	100–139	<100
**Points**	0	1	2	3
**Total CONUT score**	0–1	2–4	5–8	9–12

The CCI is a recently proposed classification for evaluating post-operative complications. This score is more sophisticated respect to the more commonly used Dindo-Clavien classification system (17). The CCI carries the advantage of capturing the burden of the entire morbidity rather than grading only the most severe complication (18). Dindo-Clavien grade I corresponds to 8.7, grade II to 20.9, grade IIIa to 26.2, grade IIIb to 33.7, grade IVa to 42.4, grade IVb to 46.2, and grade V to 100. In the liver transplantation setting, CCI has shown a good prediction ability for 90-day and 1-year graft loss risk (19). The CCI ranges from 0 (i.e., absence of post-operative complications) to 100 (i.e., death) (12, 18). A web-calculator was used for estimating CCI (available at https://www.assessurgery.com). The CCI was calculated using the following original algorithm: CCI = [√(wC1 + wC2 … + wCx)]/2.

All the complications collected were summed, even if the same patient received several times multiple administrations of the same medical (i.e., blood transfusion) or interventional (i.e., various radiological or surgical approaches) treatment. In the present study, the entire population was categorized into two groups according to the presence of a low (<42.1) or high (≥42.1) CCI value. The CCI threshold value of 42.1 was set according to previously published studies (20). The CCI value was calculated at the time of discharge after LT.

### Statistical Analysis

Continuous variables were reported as medians and interquartile ranges (IQR). Categorical variables were reported as numbers and percentages. Mann-Whitney U test and Fisher's exact test were used to compare continuous and categorical variables, respectively.

Missing data relative to study covariates always involved <10% of patients. In all the cases, missing data were handled with a single imputation method. In detail, a median of nearby-points imputation was adopted. The median instead of the mean was adopted due to the skewed distribution of the managed variables (21).

With the intent to compensate for the non-randomized design of this retrospective study, the population was “balanced” using Inverse Probability Treatment Weighting (IPTW). With the intent to perform the comparison between low and high CCI groups, twelve potential confounders were included in the model: patient age, patient male sex, HCC, hepatitis C virus (HCV) positive status, acute liver failure, waiting list duration, MELDNa, donor age, donor male sex, cold ischemia time (CIT), piggy-back caval reconstruction, cava replacement with veno-venous bypass (VVB).

With the intent to reduce the artificial increase of the sample size, and, therefore, of the type I error rate (namely, the increased number of false positives) caused by the inflated sample size in the pseudo data, we used stabilized weights (SW) according to the formula:


$$\begin{array}{l} \text{SW}=\text{p}/\text{PS for the study group};\\ \text{SW}=\;\left(\text{1}-\text{p}\right)/\left(\text{1}-\text{PS}\right)\text{for the control group} \end{array}$$

where *p* is the probability of etiology without considering covariates and PS is the propensity score.

Because *p*-values can be biased from population size, results from the comparisons between covariates subgroups were reported as effect size (*D* value): values < |0.1| indicated very small differences between means, values between |0.1| and |0.3| indicated small differences, values between |0.3| and |0.5| indicated moderate differences, and values >|0.5| indicated considerable differences (22).

A multivariable logistic regression model was developed in the post-IPTW population for the risk of CCI ≥ 42.1. Odds ratios (ORs) and 95% confidence intervals (95% CI) were reported. A backward conditional method was used for identifying the risk factors for high CCI.

The accuracy of the CONUT score was assessed for the risk of CCI ≥ 42.1 through the Harrel's c statistic. The area under the curve (AUC) and 95% CIs were reported. The model accuracy was compared with five other variables: MELDNa, MELD, D-MELD, waiting time duration, and CIT. Separate AUC of ROC curves were calculated and analyzed for comparing the single components of the CONUT score (albumin, lymphocyte, and cholesterol).

Ninety-day patient death rates were evaluated using the Kaplan-Meier method, and the log-rank test was adopted to compare the obtained survivals.

Variables with a *P* < 0.05 were considered statistically significant. We used the SPSS statistical package version 27.0 (SPSS Inc, Chicago, IL, USA) for the statistical analyses.

## Results

Two hundred and nine patients were included in the study population. The median follow-up period after LT was 37 months (IQR = 17–57). During the follow-up, 32/209 (15.3%) patients died, of whom 13 (6.2%) within 90 days from LT. In all the early deaths, the cause was a liver-specific condition (i.e., technical problems in six patients and graft failure in seven). In the late deaths, 13 patients died for liver-specific conditions (biliary complications, vascular complications, liver disease recurrence, acute rejection), while six died due to non-liver-specific conditions.

Patient characteristics are reported in Table 2. Several differences were reported between the two groups. Patients with a high-CCI value less commonly had HCC (37.9 vs. 56.3%; *P* = 0.02) and presented a median higher MELD value (21 vs. 16; *P* < 0.0001).

**Table 2:** Comparison between the Low- (<42.1) and the High-CCI (≥42.1) Group.

**Variables**	**CCI <42.1** **(*n* = 151)**	**CCI ≥42.1** **(*n* = 58)**	***P***
	**Median (IQR) or n (%)**		
**Recipient**			
Age, years	58 (51–63)	57 (47–63)	0.26
Male sex	128 (84.8)	54 (93.1)	0.17
Height, cm	170 (165–177)	175 (169–177)	0.13
Weight, kg	76 (65–87)	80 (68–89)	0.52
BMI	26 (23–29)	26 (23–29)	0.75
Waiting time duration, months	4 (1–10)	3 (0–7)	0.10
HCC	85 (56.3)	22 (37.9)	0.02
Underlying liver disease^*^			
HCV	50 (33.1)	11 (19.0)	0.06
HBV	29 (19.2)	10 (17.2)	0.84
Alcohol	59 (39.1)	21 (36.2)	0.75
NASH	32 (21.2)	15 (25.9)	0.47
Biliary cirrhosis	7 (4.6)	4 (6.9)	0.50
ALF	4 (2.6)	5 (8.6)	0.12
Other	20 (13.2)	7 (12.1)	1.00
T2DM	42 (27.8)	18 (31.0)	0.73
Requiring insulin	26 (17.2)	11 (19.0)	0.84
Arterial hypertension	32 (21.2)	9 (15.5)	0.44
CONUT	5 (3–7)	7 (5–9)	<0.0001
Albumin (g/L)	36 (31–40)	31 (26–35)	<0.0001
Total cholesterol (mg/dL)	129 (91–159)	100 (71–137)	0.001
Lymphocyte count*10^9^/L	1.03 (0.71–1.41)	0.86 (0.62–1.29)	0.09
MELD	16 (10–23)	21 (15–30)	0.001
MELDNa	18 (11–25)	23 (17–29)	0.001
D-MELD	853 (517–1,288)	1,128 (723–1,601)	0.002
**Donor**			
Age, years	58 (45–71)	63 (46–74)	0.42
Male sex	77 (51.0)	23 (39.7)	0.17
Height, cm	167 (160–175)	165 (160–171)	0.30
Weight, kg	72 (65–85)	71 (62–78)	0.37
BMI	26 (23–28)	26 (24–28)	0.95
Cause of death			
CVA	105 (69.5)	39 (67.2)	0.74
Blunt trauma	36 (23.8)	18 (31.0)	0.30
Anoxia	8 (5.3)	1 (1.7)	0.45
Other	2 (1.3)	0 (–)	1.00
T2DM	17 (11.3)	9 (15.5)	0.48
Requiring insulin	5 (3.3)	2 (3.4)	1.00
Arterial hypertension	64 (42.4)	26 (44.8)	0.76
**Transplant**			
CIT, minutes	420 (370–450)	450 (420–518)	<0.0001
Piggy-back caval reconstruction	116 (76.8)	29 (50.0)	<0.0001
Temporary portocaval shunt	28 (18.5)	13 (22.4)	0.56
Cava replacement with VVB	6 (4.0)	14 (24.1)	<0.0001

* *In some cases, more liver diseases were present contemporaneously.*

*CCI, comprehensive complication index; IQR, interquartile ranges; n, number; BMI, body mass index; HCC, hepatocellular cancer; HCV, hepatitis C virus; HBV, hepatitis B virus; NASH, non-alcoholic steato-hepatitis; ALF, acute liver failure; T2DM, type 2 diabetes mellitus; CONUT, Controlling Nutritional Status; MELD, model for end-stage liver disease; Na, sodium; D-MELD, donor-MELD; CVA, cerebrovascular accident; CIT, cold ischemia time; VVB, veno-venous bypass*.

**Figure 1:**
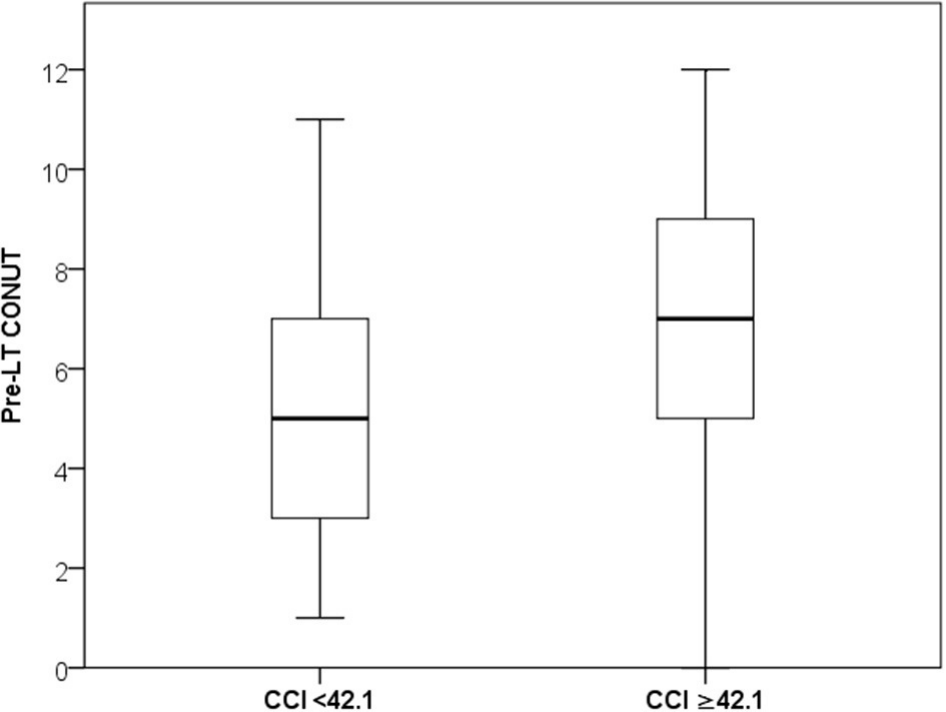
Median and IQR values of CONUT in the low- and high-CCI groups.

In all the cases, the median values of the CONUT score variables were lower in the high-CCI patients. In detail, median albumin value was 31 vs. 36 g/L (*P* < 0.0001), median total cholesterol was 100 vs. 129 mg/dL (*P* = 0.001), and median lymphocyte count was 0.86 vs. 1.03^*^10^9^/L (*P* = 0.09). Consequently, the CONUT score value was significantly superior in the high-CCI group (median: 7 vs. 5; *P* < 0.0001) (Figure 1).

No statistical differences were observed concerning the donor characteristics. As for the transplant surgical procedure, the CIT was longer in the high-CCI group (450 vs. 420 min; *P* < 0.0001). Piggy-back caval anastomosis was observed less commonly in the high-CCI group (50.0 vs. 76.8%; *P* < 0.0001), with higher usage of cava replacement with veno-venous bypass (24.1 vs. 4.0; *P* < 0.0001).

**Figure 2:**
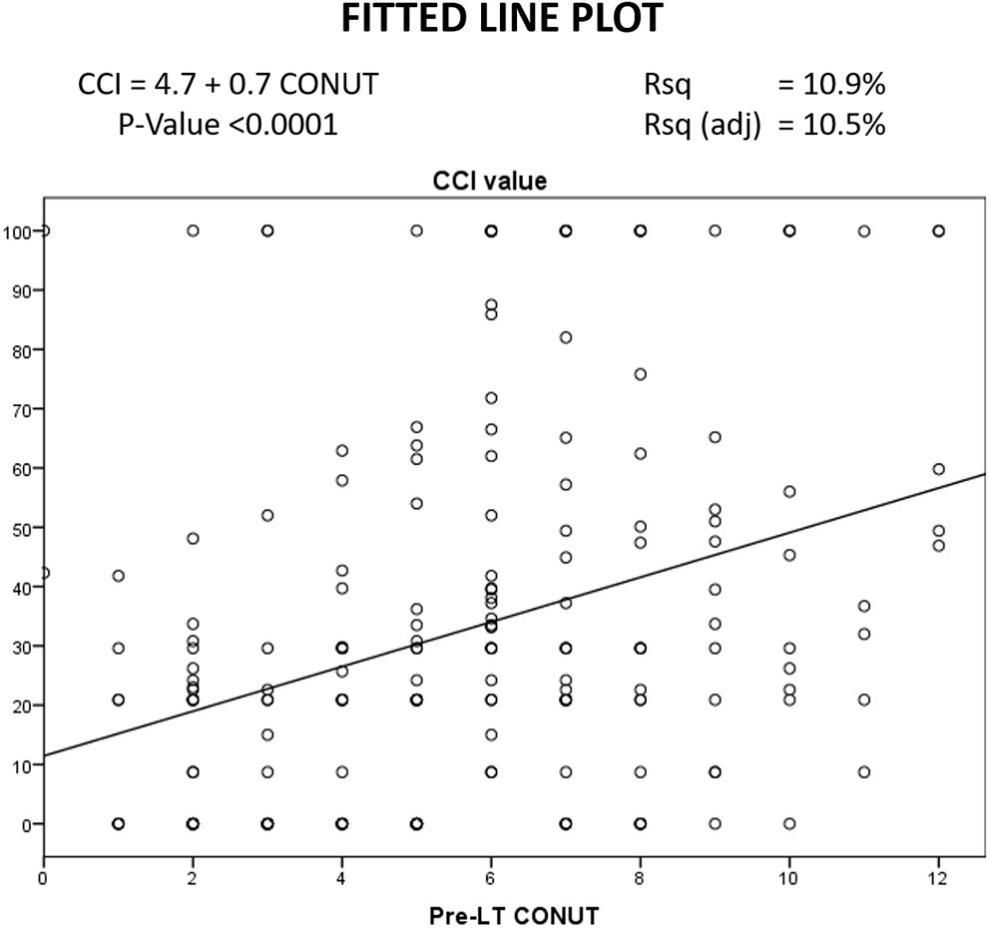
Linear correlation between CONUT and CCI.

A linear correlation was reported between the CCI and the CONUT values, suggesting a potential connection between these two variables. In detail, a statistical significance was observed (*P* < 0.0001), although the adjusted R squared value showed low values (10.5%) (Figure 2).

To eliminate potential confounders, the two groups were “balanced” for twelve variables emerging as significantly different between the groups. A stabilized IPTW allowed to reduce the initial differences. As reported in Table 3, also variables initially showing relevant differences of the means such as MELDNa (*D*-value 0.51), CIT (*D*-value 0.63), and piggy-back caval reconstruction (*D*-value −0.55), all showed small or very small differences after the IPTW. Thanks to the use of a stabilized approach, the sample size of the pseudo population did not significantly differ with respect to the initial unbalanced population (212 vs. 209 cases).

**Table 3:** Effect of IPTW on the variables used for balancing the two groups.

	**Cohen's** ***D*****-value**	
	**Pre-IPTW**	**Post-IPTW**
Recipient age	−0.19	−0.07
Recipient male sex	0.29	−0.19
Waiting time duration, months	−0.12	0.06
HCC	−0.37	−0.10
HCV	−0.34	−0.02
ALF	0.23	−0.02
MELDNa	0.51	0.13
Donor age	0.09	−0.08
Donor male sex	−0.23	−0.24
CIT	0.63	−0.15
Piggy-back caval reconstruction	−0.55	−0.05
Cava replacement with VVB	0.09	−0.02

*IPTW, inverse probability therapy weighting; HCC, hepatocellular cancer; HCV, hepatitis C virus; ALF, acute liver failure; MELDNa, model for end-stage liver disease sodium; CIT, cold ischemia time; VVB, veno-venous bypass*.

The risk factors for a CCI ≥42.1 were investigated in the post-IPTW population using multivariable logistic regression. Twelve different potential risk factors were initially introduced in the mathematical model. Using a backward Wald method, only the CONUT score before LT was an independent risk factor for high CCI. In detail, CONUT showed an OR = 1.39 (95%CI = 1.21–1.58; *P* < 0.0001). In other terms, each increase of one point of CONUT score increased the risk of high CCI by 39% (Table 4). All the other patient-, donor-, and transplant-related variables failed to have a relevant role as risk factors for high CCI.

**Table 4:** Multivariable logistic regression model for the risk of CCI ≥42.1.

**Variable**	**Beta**	**SE**	**Wald**	**OR**	**95%CI**		**P**
					**Lower**	**Upper**	
Pre-LT CONUT	0.33	0.07	23.29	1.39	1.21	1.58	<0.0001
Donor male sex	−0.54	0.34	2.42	0.59	0.30	1.15	0.12
WT duration in months	0.03	0.02	2.31	1.03	0.99	1.07	0.13
Donor age	−0.004	0.01	0.22	0.996	0.98	1.01	0.64
Recipient age	0.003	0.02	0.04	1.003	0.97	1.04	0.84
Recipient male sex	−0.06	0.44	0.02	0.94	0.40	2.21	0.89
Constant	−2.89	1.24	5.43	0.06	–	–	0.02

*Variables initially introduced into the model: recipient age, recipient male sex, HCC, HCV, ALF, WT duration, CONUT, donor age, donor male sex, MELDNa, CIT, Piggy-back caval reconstruction.*

*SE, standard error; OR, odds ratio; CI, confidence intervals; CONUT, Controlling Nutritional Status; WT, waiting time; HCC, hepatocellular cancer; HCV, hepatitis C virus; ALF, acute liver failure; MELDNa, model for end-stage liver disease sodium; CIT, cold ischemia time*.

**Figure 3:**
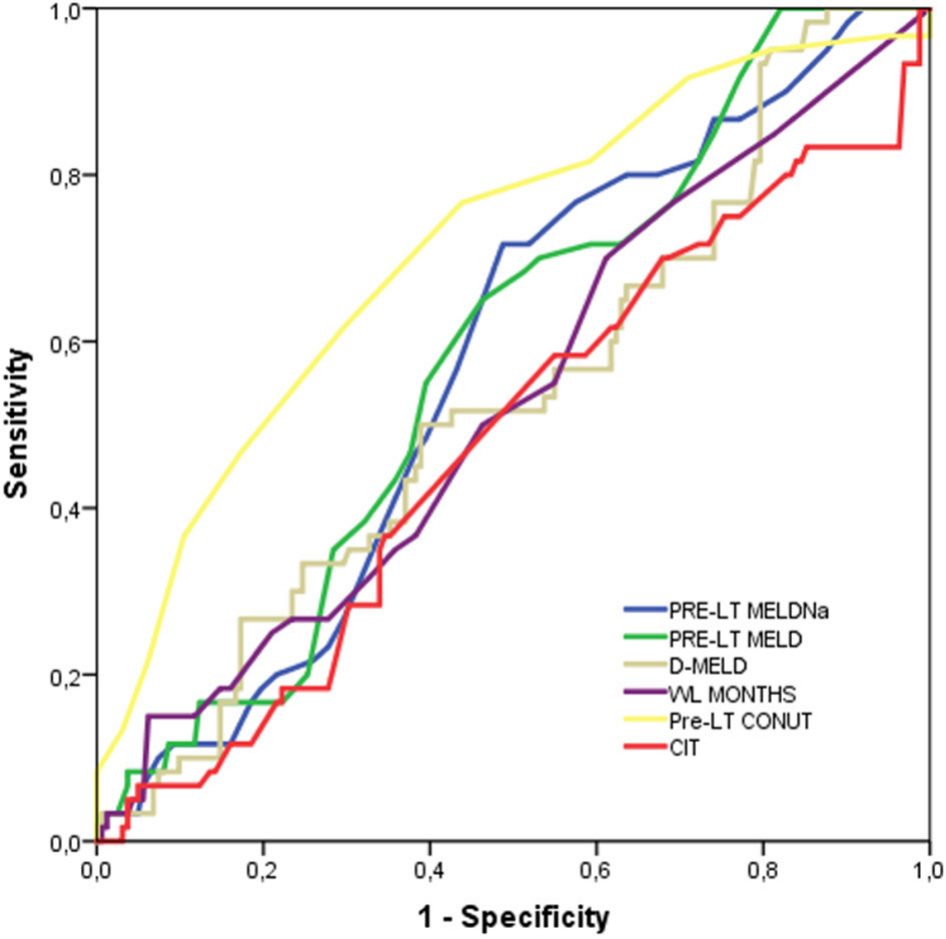
Harrel's c-statistics for the diagnosis of CCI ≥42.1.

At c-statistics analysis in the pseudo population, the CONUT score was the unique tested variable showing diagnostic ability, with an AUC = 0.72 (95%CI = 0.64-0.79; *P* < 0.0001). All the other potential diagnostic tools measured at the time of LT (i.e., MELD, MELDNa, D-MELD) failed to predict a high CCI, as reported in Table 5 and Figure 3. All of the single variables of the CONUT score showed significant AUC in terms of prognostic ability for the risk of CCI ≥42, in particular albumin AUC was superimposable to CONUT score AUC (data not shown).

**Table 5:** C-statistics for the evaluation of CONUT performance for the diagnosis of CCI ≥42.1.

**Variables**	**AUC**	**SE**	**95%CI**		**P**
			**Lower**	**Upper**	
CONUT	0.72	0.04	0.64	0.79	<0.0001
MELD	0.58	0.04	0.50	0.66	0.06
MELDNa	0.57	0.04	0.49	0.65	0.09
D-MELD	0.53	0.04	0.45	0.62	0.44
WT duration	0.53	0.04	0.44	0.61	0.51
CIT	0.48	0.04	0.39	0.57	0.65

*AUC, area under the curve; SE, standard error; CI, confidence intervals; CONUT, Controlling Nutritional Status; MELD, model for end-stage liver disease; Na, sodium; D-MELD, donor-MELD; WT, waiting time; CIT, cold ischemia time*.

**Figure 4:**
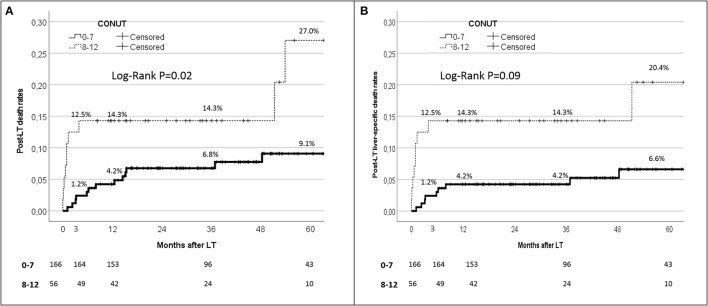
Kaplan-Maier analysis for the 90-day patient survival rates in patient initially with CONUT 0–7 vs 8–12.

When the post-IPTW population was split according to the CONUT value, the 90-day, 1-year, and 5-year patient death rates were 1.2, 4.2, and 9.1%, respectively, when the CONUT value was <8. On the opposite, when a CONUT score ≥8 was observed, the 90-day, 1-year, and 5-year patient death rates increased to 12.5, 14.3, and 27.0%, with a statistically significant difference between the two subgroups (log-rank *P* = 0.02) (Figure 4A).

Similar results were observed when only the liver-specific death rates were reported (Figure 4B). In detail, the 90-day, 1-year, and 5-year liver-specific death rates were 1.2, 4.2, and 6.6%, respectively, with CONUT scores <8, whilst they increased to 12.5, 14.3, and 20.4% respectively with CONUT scores ≥8. Also in this case, a statistically significant difference between the two subgroups was reported (log-rank *P* = 0.09).

## Discussion

Malnutrition and immunologic compromise increase the risk of post-LT complications, particularly after “extended-criteria donor to frail recipient” matches (23, 24). The possibility to pre-operatively predict this potential risk is pivotal for optimizing resource allocation and preserving LT outcomes.

Our study demonstrated the efficacy of the CONUT score in predicting severe post-LT complications, with an OR = 1.39. Moreover, patients with high (≥8) pre-transplant CONUT values showed poor post-operative 90-day as well as long-term patient survival rates. In particular, we encountered a higher rate of liver-specific deaths at all the time points analyzed, highlighting the role of the pre-transplant condition of the recipient.

Our findings align with previous studies exploring the predictive role of the CONUT score in the setting of hepatic (10, 25–27), thoracic, urological, and gastrointestinal oncological surgery (28–32). A study from China (*N* = 94) showed that pre-operative CONUT was the best predictor of overall and recurrence-free survivals in patients resected for hilar cholangiocarcinoma (10).

A multicenter study from Japan (*N* = 2461) similarly showed that the pre-operative CONUT score was predictive of worse overall and recurrence-free survivals in patients resected for HCC, even after propensity score matching analysis (27).

A study from Japan (*N* = 204) suggested that the CONUT score was a strong independent predictor of survival among stage II/III colorectal cancer patients (28).

As for the setting of LT, only a limited number of studies have been published (6, 33).

A study from Italy (*N* = 280) explored the specific impact of CONUT in the LT population with HCC. Of relevance, this study failed to observe any correlation between the CONUT score and post-LT poor survival or tumor recurrence (6). A potential explanation for these results could derive from the super-selection of the explored population. In fact, in LT, HCC patients represent a well-known selected population with a more compensated liver condition and, therefore, a predictable narrower spread of CONUT values.

Another study from the same authors (*N* = 324) investigated the post-LT trend of CONUT in HCC patients, reporting worse values in the early post-LT period than the pre-LT values and a substantial improvement after the post-LT third month (33).

Concerning the previously published studies exploring the role of the CONUT score in LT, our study presents some beneficial aspects.

As an example, our analysis was performed on HCC patients and patients with an acute or a severe chronic end-stage liver disease (ESLD). As well known, ESLD causes a reduction in the biosynthetic activity of the liver, translating into lower levels of circulating proteins such as albumin and apolipoproteins. Consequently, the CONUT score reflects the actual liver functional reserve, being particularly useful in the specific setting of patients with more advanced liver disease (34). As a confirmation of this datum, we observed higher CONUT values and lower median levels of cholesterol and albumin in the high CCI group, namely the group comprising more advanced ESLD cases. When comparing the single components of the CONUT score, albumin showed higher predictive ability compared to cholesterol and lymphocyte count, in regards of post-LT morbidity. Similar findings were obtained in the field of thoracic oncologic surgery, with albumin and CONUT having nearly superimposable AUC values, superior to both cholesterol and lymphocyte count AUCs (35).

Another critical aspect to underline is the statistical approach we adopted with the intent to minimize confounding phenomena. Several potential confounders have been identified to bias our results when we compared the two groups with low or high CCI. For example, patients with a lower CCI were more likely to have HCC and a lower median MELD score (i.e., less severe liver disease). Conversely, patients with a higher CCI presented a longer CIT, potentially caused by the increased complexity and longer duration of the hepatectomy (i.e., more complex surgery due to severe liver disease). Thanks to the use of a stabilized IPTW, we were able to “balance” our population for these potential confounders, therefore eliminating the potential bias caused by their effect.

Interestingly, no statistical difference was detected concerning the donor characteristics even before using the IPTW, further emphasizing the prominent role of the initial ESLD severity in determining post-LT complications. However, no firm assumptions can be drawn in these regards due to the abovementioned limitations.

Another relevant aspect of the present study was that the diagnostic performance of the CONUT score in predicting severe post-LT complications was compared for the first time with other commonly used diagnostic tools for organ allocation and donor-recipient match, namely MELD, MELDNa, and D-MELD score. Interestingly, the CONUT score had the best performance as a pre-operative diagnostic tool for predicting a poor post-LT course. The availability of a tool to predict complications is highly desirable and is a topic of central interest in the transplant community, as the complexity of LT procedures continues to increase and more malnourished and immunocompromised patients are evaluated. Similarly to other fields of application, our results confirm the advantages of the CONUT score as a cheap, user-friendly, and pre-operatively available score based on routine blood tests.

Moreover, the pre-transplant CONUT values should consent to target high-risk patients, offering interventions that tackle frailty and sarcopenia before LT (e.g., using nutritional supplementation, immunomodulation, exercise) (36, 37).

As an example, a recent study from Italy reported that an “urgency” model combining MELDNa and sarcopenia should be used to prioritize the sarcopenic patients with an initial MELDNa <20 on the list, further underlying the relevance of the nutritional status in the LT candidates and the scarce ability of the MELD system in capturing the actual complexity of these patients (38).

The use of rehabilitation programs based on multidisciplinary “training” to enhance physical strength and nutritional status has been proven to increase the physiologic reserve before surgery and withstand complications after transplant (39).

The importance of these considerations is even more critical in light of the evolving epidemiology of LT candidates due to the increased prevalence of non-alcoholic steatohepatitis (NASH). A recent study investigating the relationship between frailty and cirrhosis etiology revealed that NASH patients were the frailest category of LT candidates, justifying particular attention to the liver functional reserve and malnourishment and immunologic impairment when a patient is transplanted (40).

The CONUT score should play an important role also in the evaluation of the post-LT course, due to the modification of its value in the months after the transplant (33). In this setting, immunosuppression might play a relevant role, mainly impacting on some of the variables composing the CONUT score (e.g., mTOR inhibitors and cholesterol). Further studies are required for the validation of post-transplant CONUT score as a prognosticator of long-term outcomes.

Our study has some limitations. Since this was a retrospective study, the time-point of data collection before LT was heterogeneous. To minimize this heterogeneity, we decided to consider only the blood tests available two months before the transplant. Such a decision impacted the global number of patients we were able to enroll for the study. Many patients transplanted during the study period were not included in the analysis because of outdated tests. The main problem was connected with the cholesterol test, which was not routinely repeated during the LT waitlist. However, despite the consequent sample size reduction, we thought it was a more severe bias to use CONUT calculations based on outdated blood tests (for example, at the time of waiting list inscription), therefore losing the ability of the score to capture the actual nutritional status of the patients at the time of transplantation.

Another already reported limiting factor relates to the number of patients included. Considering our sample size, we were aware that a selection bias could jeopardize the quality of the results of our study. To mitigate such risk, we chose the stabilized IPTW, which allowed us to minimize the effect of potential confounders.

A potential limit to report is that some albumin levels could be partly increased by the intravenous supplementation administered to decompensated ESLD patients. Such practice has been become routine since the supplementation of intravenous human albumin solution was demonstrated to titrate the higher level of prostaglandin PGE2 that is responsible for the macrophage impairment in patients with acutely decompensated cirrhosis (41).

However, only a limited number of patients (<10%) in our series present such a condition. Moreover, other commonly used scores carry similar problems (i.e., MELD and plasma infusion). Thus, we considered this limitation unresolvable in the clinical practice and only marginally impacting on the observed results.

Lastly, due to the lack of sufficient data, we could not investigate the importance of decreasing HDL levels in relation to total cholesterol. It has been observed that HDL levels tend to drop proportionally with the evolution of the severity of the ESLD (42). Further studies are needed to investigate if HDL cholesterol levels might further refine the CONUT score in predicting post-LT outcomes.

In conclusion, our study shows a correlation between the CONUT score and the development of severe complications and 90-day as well as long-term mortality after liver transplantation. The CONUT score proved to be a reliable and easy-to-calculate tool that could be integrated in clinical practice with affordable extra costs. Prospective studies are required to corroborate the present findings.

## Data Availability Statement

The raw data supporting the conclusions of this article will be made available by the authors, without undue reservation.

## Ethics Statement

Ethical review and approval was not required for the study on human participants in accordance with the local legislation and institutional requirements. Written informed consent for participation was not required for this study in accordance with the national legislation and the institutional requirements.

## Author Contributions

GS and QL: conception and design. SA and MR: administrative support. SA, MR, and AWA: provision of study materials or patients. FF, GB, AM, and QL: collection and assembly of data. QL, AWA, and GS: data analysis and interpretation. All authors: manuscript writing and final approval of manuscript.

## Conflict of Interest

The authors declare that the research was conducted in the absence of any commercial or financial relationships that could be construed as a potential conflict of interest.

## Publisher's Note

All claims expressed in this article are solely those of the authors and do not necessarily represent those of their affiliated organizations, or those of the publisher, the editors and the reviewers. Any product that may be evaluated in this article, or claim that may be made by its manufacturer, is not guaranteed or endorsed by the publisher.
